# Supplementary material for: Mechanistic insight into TRIP13-catalyzed Mad2 structural transition and spindle checkpoint silencing
Source: Nat Commun. 2017 Dec 5;8:1956. doi: 10.1038/s41467-017-02012-2 (PMC5717197; doi:10.1038/s41467-017-02012-2)
Supplement: Supplementary file 1 — Supplementary Information [file 41467_2017_2012_MOESM1_ESM.pdf]

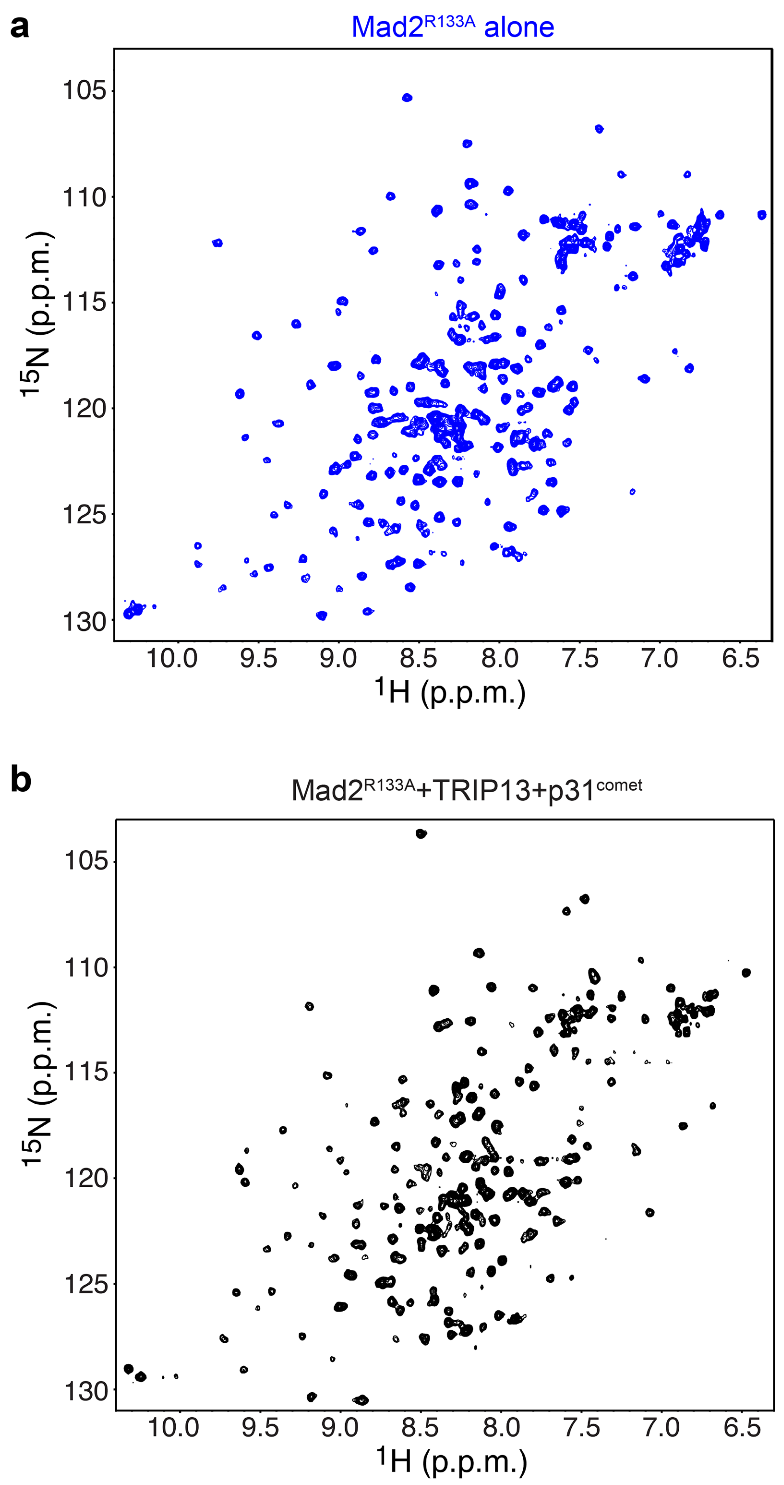

Supplementary Figure 1 | TRIP13 catalyzes the conversion of C-Mad2 to O-Mad2 with the help of p31^{comet}. a The ^{1}H-^{15}N HSQC spectrum of ^{15}N-Mad2^{R133A} before the addition of TRIP13 and ΔN35-p31^{comet}. **b** The ^{1}H-^{15}N HSQC spectrum of ^{15}N-Mad2^{R133A} after the addition of ATP and sub-stoichiometric amounts of TRIP13 and ΔN35-p31^{comet}

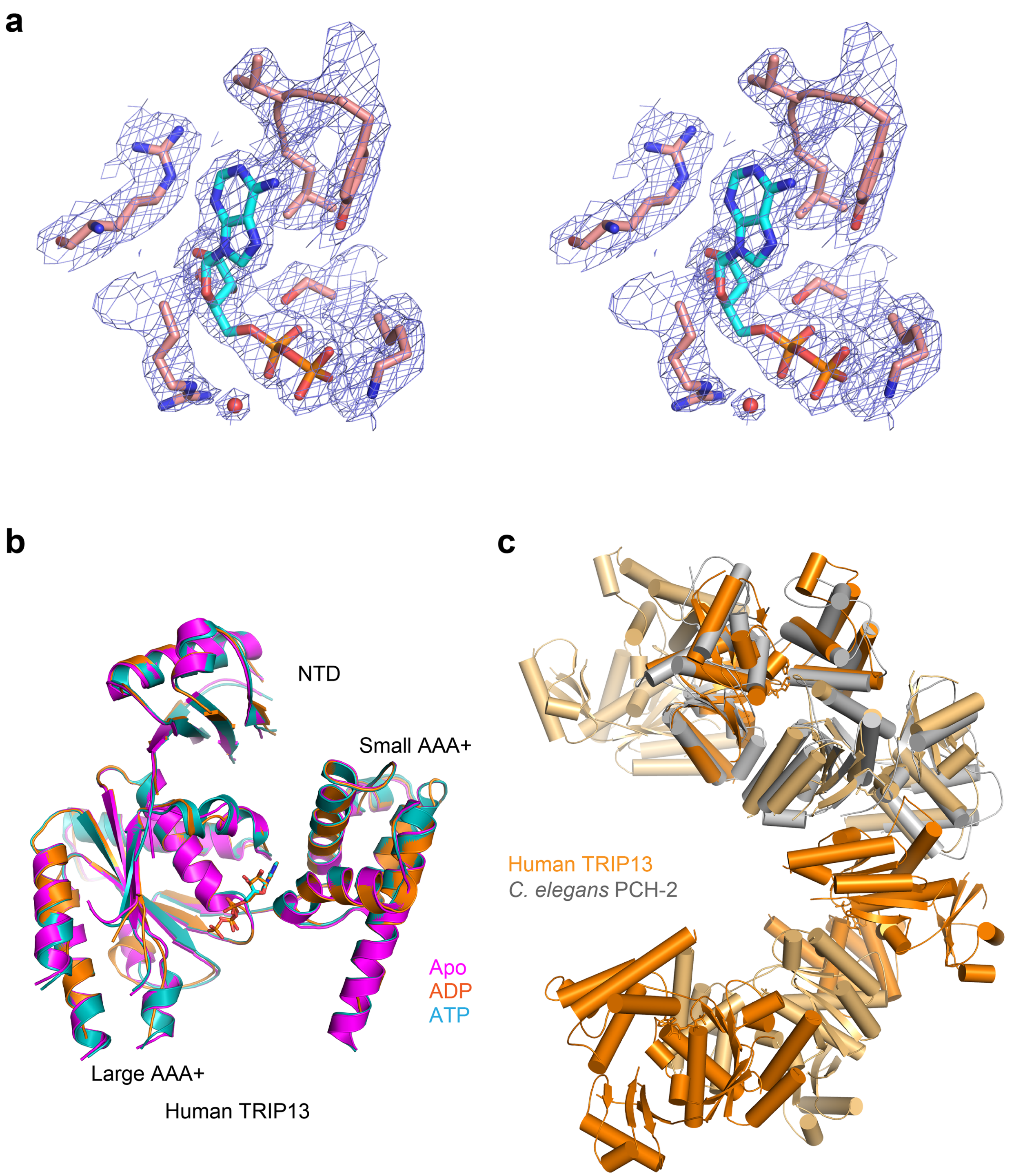

Supplementary Figure 2 | Structure of human TRIP13. **a** Stereo view of the 2Fo-Fc electron density map (contoured at 1 σ) of ADP and ADP-binding residues of human TRIP13. **b** Overlay of the ribbon diagrams of the structures of human TRIP13 bound to ADP (orange; this study), bound to ATP (cyan), and in nucleotide-free state (magenta), with the bound nucleotides shown in sticks. **c** Ribbon diagram of a human TRIP13 hexamer observed in the crystal, with alternating TRIP13 monomers colored orange and light orange. Two PCH-2 protomers in the closed conformation are overlaid onto this TRIP13 assembly and shown in gray

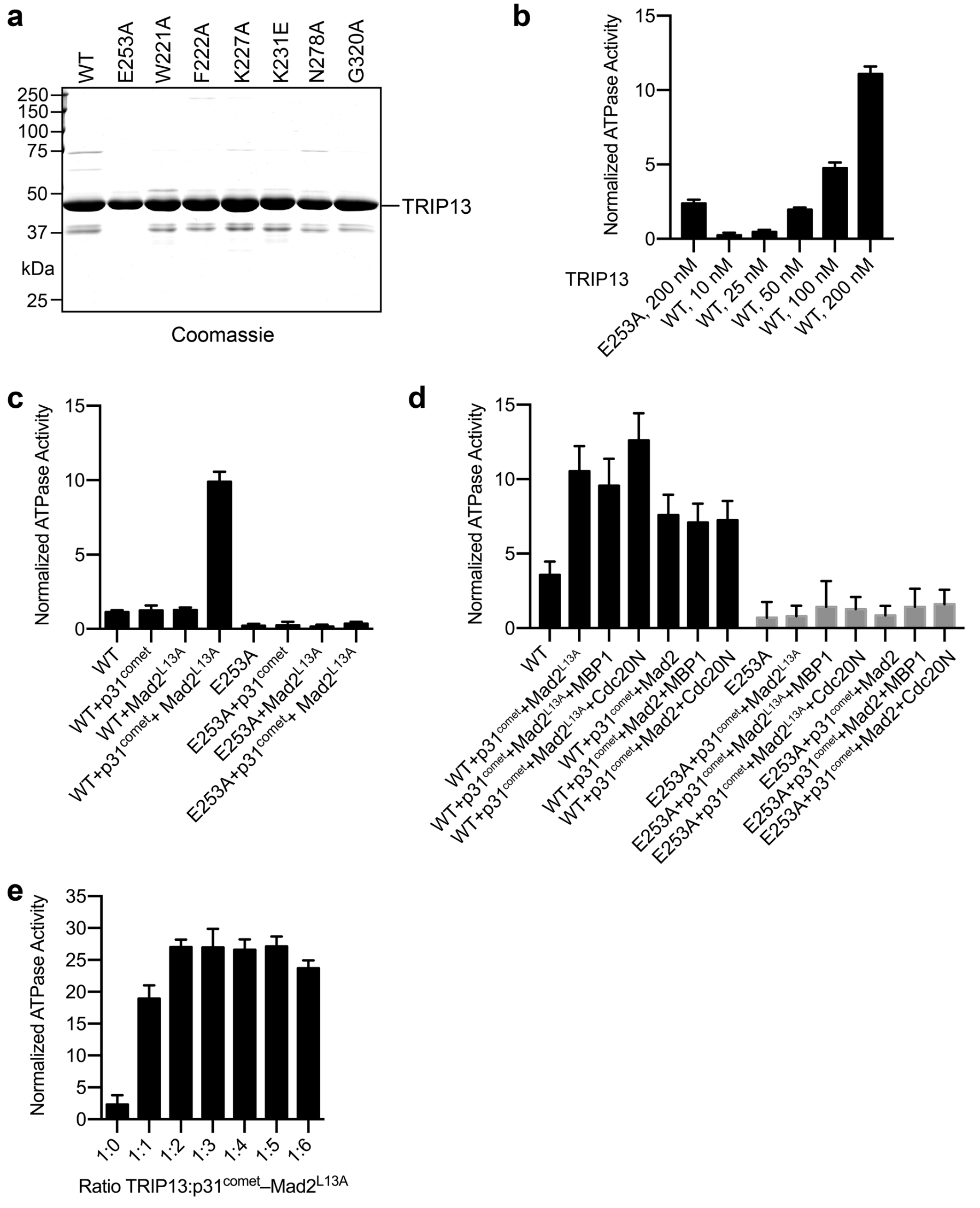

Supplementary Figure 3 | Development of a high-throughput assay to assess the ATPase activity of TRIP13 mutants. **a** Coomassie-stained SDS-PAGE gel of recombinant purified TRIP13 wild type (WT) and the indicated mutants. **b** Normalized ATPase activities of TRIP13 WT and TRIP13 E253A at the indicated concentrations. Mean ± SD; n = 3. **c** Stimulation of the ATPase activity of TRIP13 (25 nM) by ΔN35-p31^{comet} (50 nM), Mad2^{L13A} (50 nM), or the ΔN35-p31^{comet}–Mad2^{L13A} complex (50 nM). Mean ± SD; n = 10. **d** Stimulation of the ATPase activity of TRIP13 (25 nM) by ΔN35-p31^{comet} (125 nM) and different forms of C-Mad2, including Mad2^{L13A} (125 nM), dimeric Mad2 (125 nM), Mad2–MBP1 (125 nM), or Mad2–Cdc20N (125 nM). Mean ± SD; n = 10. **e** Stimulation of the ATPase activity of TRIP13 (25 nM) by ΔN35-p31^{comet}–Mad2^{L13A} at the indicated molar ratios. Mean ± SD; n = 4

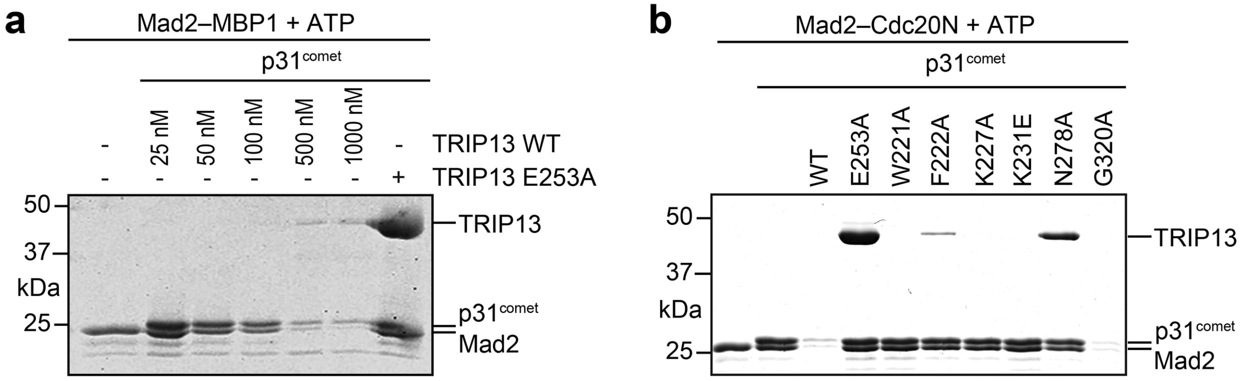

Supplementary Figure 4 | TRIP13 promotes dissociation of Mad2 from peptide ligands. **a** MBP1-coupled beads were first incubated with Mad2 and then incubated with 1 μM TRIP13 E253A or WT at the indicated concentrations in the presence of 2 μM ΔN35-p31^{comet} and 1 mM ATP. Proteins bound to beads were analyzed by SDS-PAGE and stained with Coomassie blue. **b** Cdc20N-coupled beads were first incubated with Mad2 and then incubated with TRIP13 wild type (WT) and the indicated mutants (500 nM) in the presence of ΔN35-p31^{comet} (1 μM) and ATP (1 mM). Proteins bound to beads were analyzed by SDS-PAGE and stained with Coomassie blue

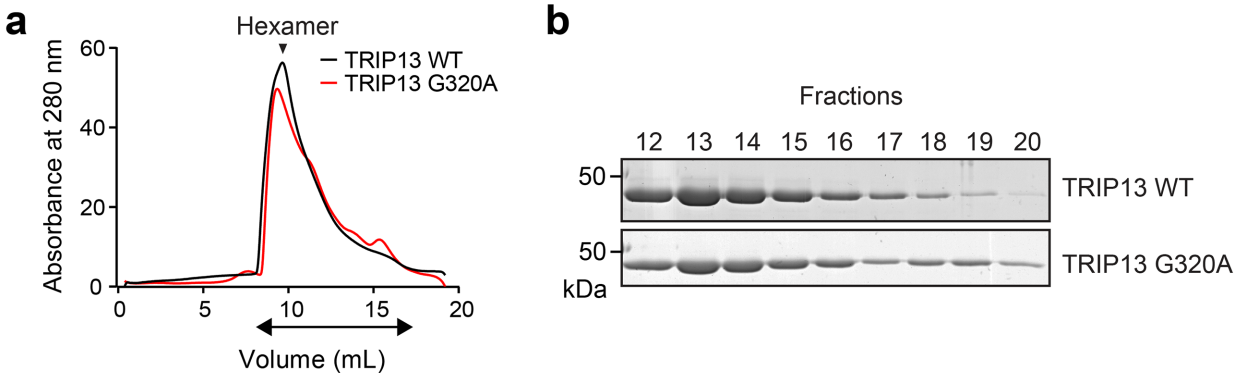

Supplementary Figure 5 | TRIP13 G320A forms oligomers in the presence of ATP. **a** UV traces of TRIP13 wild type (WT) and G320A fractionated on a gel filtration column. **b** Protein-containing fractions in **a** were analyzed by SDS-PAGE and stained with Coomassie

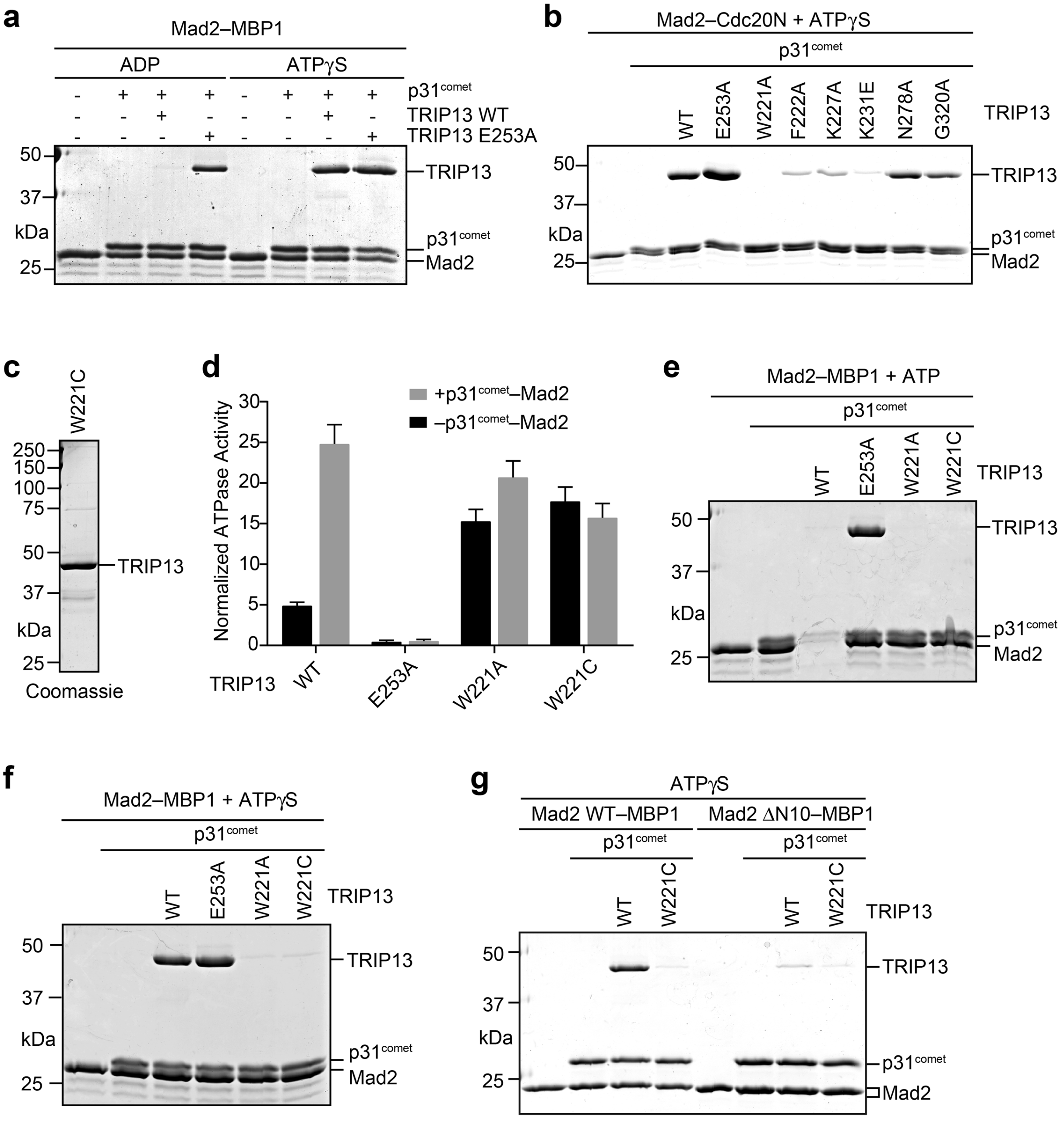

Supplementary Figure 6 | The p31^{comet}–Mad2 complex prefers to bind to TRIP13 in the ATP-bound state. **a** MBP1-coupled beads were first incubated with Mad2 and then incubated with 500 nM TRIP13 wild type (WT) or E253A and 1 μM ΔN35-p31^{comet} in the presence of 1 mM ADP or ATPγS. Proteins bound to beads were analyzed by SDS-PAGE and stained with Coomassie blue. **b** Cdc20N-coupled beads were first incubated with Mad2, and then incubated with TRIP13 wild type (WT) or the indicated mutants (500 nM), ΔN35-p31^{comet} (1 μM), and ATPγS (1 mM). Proteins bound to beads were analyzed by SDS-PAGE and stained with Coomassie blue. **c** Coomassie-stained SDS-PAGE gel of recombinant purified TRIP13 W221C. **d** ATPase activities of the indicated TRIP13 proteins at 25 nM with (+) or without (–) 50 nM ΔN35-p31^{comet}–Mad2^{L13A}. Mean ± SD; n = 9. **e** MBP1-coupled beads were first incubated with

Mad2 and then incubated with TRIP13 wild type (WT) and the indicated mutants (500 nM) in the presence of ΔN35-$p31^{comet}$ (1 μM) and ATP (1 mM). Proteins bound to beads were analyzed by SDS-PAGE and stained with Coomassie blue. **f** MBP1-coupled beads were first incubated with Mad2, and then incubated with TRIP13 wild type (WT) or the indicated mutants (500 nM), ΔN35-$p31^{comet}$ (1 μM), and ATPγS (1 mM). Proteins bound to beads were analyzed by SDS-PAGE and stained with Coomassie blue. **g** MBP1-coupled beads were first incubated with Mad2 or ΔN10-Mad2, and then incubated with TRIP13 wild type (WT) (500 nM), ΔN35-$p31^{comet}$ (1 μM), and ATPγS (1 mM). Proteins bound to beads were analyzed by SDS-PAGE and stained with Coomassie blue

Supplementary Table 1 | Data collection and refinement statistics

	Native TRIP13
**Data collection**	
Space group	$P6_5$
Cell dimensions	
a, *b*, *c* (Å)	98.37, 98.37, 122.11
α, β, γ (°)	90.00, 90.00, 120.00
Wavelength (Å)	0.97918
Resolution (Å)	2.50
R_{sym}	0.079 (1.485)
$I / \sigma I$	40.6 (2.0)
Completeness (%)	100.0 (99.9)
Redundancy	15.2 (10.9)
**Refinement**	
Resolution (Å)	32.20–2.50 (2.61–2.50)
No. reflections	21,458 (1,349)
R_{work} / R_{free} (%)	18.8 (23.7) / 22.6 (29.4)
No. atoms	
Protein	2,895
Ligand/ion	27
Water	138
B-factors (Å^2)	
Protein	48.0
Ligand/ion	26.0
Water	43.6
r.m.s deviations	
Bond lengths (Å)	0.003
Bond angles (°)	0.615

*Values in parentheses are for highest-resolution shell.
